# Inhibition of major histocompatibility complex-I antigen presentation by sarbecovirus ORF7a proteins

**DOI:** 10.1073/pnas.2209042119

**Published:** 2022-09-22

**Authors:** Fengwen Zhang, Trinity M. Zang, Eva M. Stevenson, Xiao Lei, Dennis C. Copertino, Talia M. Mota, Julie Boucau, Wilfredo F. Garcia-Beltran, R. Brad Jones, Paul D. Bieniasz

**Affiliations:** ^a^Laboratory of Retrovirology, The Rockefeller University, New York, NY 10065;; ^b^Infectious Disease Division, Weill Cornell Medicine, New York, NY; 10021;; ^c^Ragon Institute of MGH, MIT, and Harvard, Cambridge, MA 02139;; ^d^Department of Pathology, Massachusetts General Hospital, Boston, MA 02114;; ^e^Howard Hughes Medical Institute, The Rockefeller University, New York, NY 10065

**Keywords:** Sarbecovirus, SARS-CoV-2, ORF7a, MHC-I down-regulation, Peptide loading complex

## Abstract

Viruses employ a variety of strategies to escape or counteract immune responses, including depletion of cell surface major histocompatibility complex class I (MHC-I), that would ordinarily present viral peptides to CD8^+^ cytotoxic T cells. As part of a screen to elucidate biological activities associated with individual severe acute respiratory syndrome coronavirus-2 (SARS-CoV-2) viral proteins, we found that ORF7a reduced cell surface MHC-I levels by approximately fivefold. Nevertheless, in cells infected with SARS-CoV-2, surface MHC-I levels were reduced even in the absence of ORF7a, suggesting additional mechanisms of MHC-I down-regulation. ORF7a proteins from a sample of sarbecoviruses varied in their ability to induce MHC-I down-regulation and, unlike SARS-CoV-2, the ORF7a protein from SARS-CoV lacked MHC-I downregulating activity. A single amino acid at position 59 (T/F) that is variable among sarbecovirus ORF7a proteins governed the difference in MHC-I downregulating activity. SARS-CoV-2 ORF7a physically associated with the MHC-I heavy chain and inhibited the presentation of expressed antigen to CD8^+^ T cells. Specifically, ORF7a prevented the assembly of the MHC-I peptide loading complex and caused retention of MHC-I in the endoplasmic reticulum. The differential ability of ORF7a proteins to function in this way might affect sarbecovirus dissemination and persistence in human populations, particularly those with infection- or vaccine-elicited immunity.

## Significance

As part of the adaptive immune response, virus-infected cells present virus-derived peptides to cytotoxic T cells on the surface of infected cells, using a protein complex termed major histocompatibility complex class-I. Some viruses counteract antigen presentation by depleting major histocompatibility complex class-I from the cell surface. We show that severe acute respiratory syndrome coronavirus-2 (SARS-CoV-2) uses an accessory protein ORF7a to interfere with the formation of peptide-major histocompatibility complex class-I complexes, prevent their movement to the cell surface, and thus inhibit antigen presentation to cytotoxic T cells. ORF7a proteins from SARS-CoV-2-related viruses vary in their ability to interfere with antigen presentation, which might affect the ability of vaccine- or infection-elicited immune responses to protect against this family of pandemic threat viruses.

To replicate and propagate in a host population that presents an immunologically hostile environment, viruses typically employ a variety of strategies to escape or counteract immune responses. Severe acute respiratory syndrome coronavirus-2 (SARS-CoV-2), a member of the sarbecovirus subgenus, has been shown to antagonize the innate immune response through the action of viral proteins (1–3) and to escape humoral immunity through variation in the neutralizing epitopes of the spike protein (4–8). Evasion of cell-mediated immunity is accomplished by many viruses through the down-regulation of surface expression of major histocompatibility complex-I, that would ordinarily present viral peptides to CD8^+^ cytotoxic T cells (9–11). For example, HIV-1 renders the virus-infected cells less visible to CD8^+^ T cells through Nef-induced endocytosis of MHC-I from the cell surface (12). In general, viruses from other families that are associated with chronic infections employ diverse mechanisms to deplete MHC-I from infected cell surfaces (10, 11). However, viruses associated with short-term acute infection do not typically induce MHC-I down-regulation.

The ∼30-kb SARS-CoV-2 genome encodes structural proteins (E, M, N, and S), nonstructural proteins (nsp1 to nsp16), and several “accessory” open reading frames (ORF3a, ORF6, ORF7a, ORF8, ORF10, ORF3b, ORF9b, and ORF9c) (13, 14). Analysis of coronavirus-host protein–protein interactions, using affinity- or proximity-based approaches, has suggested that several viral proteins (ORF3a, ORF3b, ORF7a, ORF8, M, and nsp4) associate with host proteins that are enriched in endoplasm reticulum (ER) or Golgi, the organelles where viral peptides are loaded onto MHC-I molecule and transported to the cell surface for presentation to CD8^+^ T cells (14, 15). Moreover, some reports have indicated that SARS-CoV-2 ORF8 reduces expression of MHC-I on the surface of infected cells (16, 17). Here, we show that SARS-CoV-2 ORF7a can inhibit antigen presentation by preventing the assembly of the MHC-I peptide loading complex and causing retention of MHC-I in the endoplasmic reticulum. Notably, ORF7a proteins from a sample of sarbecoviruses vary in their ability to induce MHC-I down-regulation, and a single amino acid that is variable among sarbecovirus ORF7a proteins governs the differential ability to induce in MHC-I down-regulation.

## Results

### SARS-CoV-2 ORF7a Reduces Cell Surface MHC-I Levels.

**Fig. 1.**
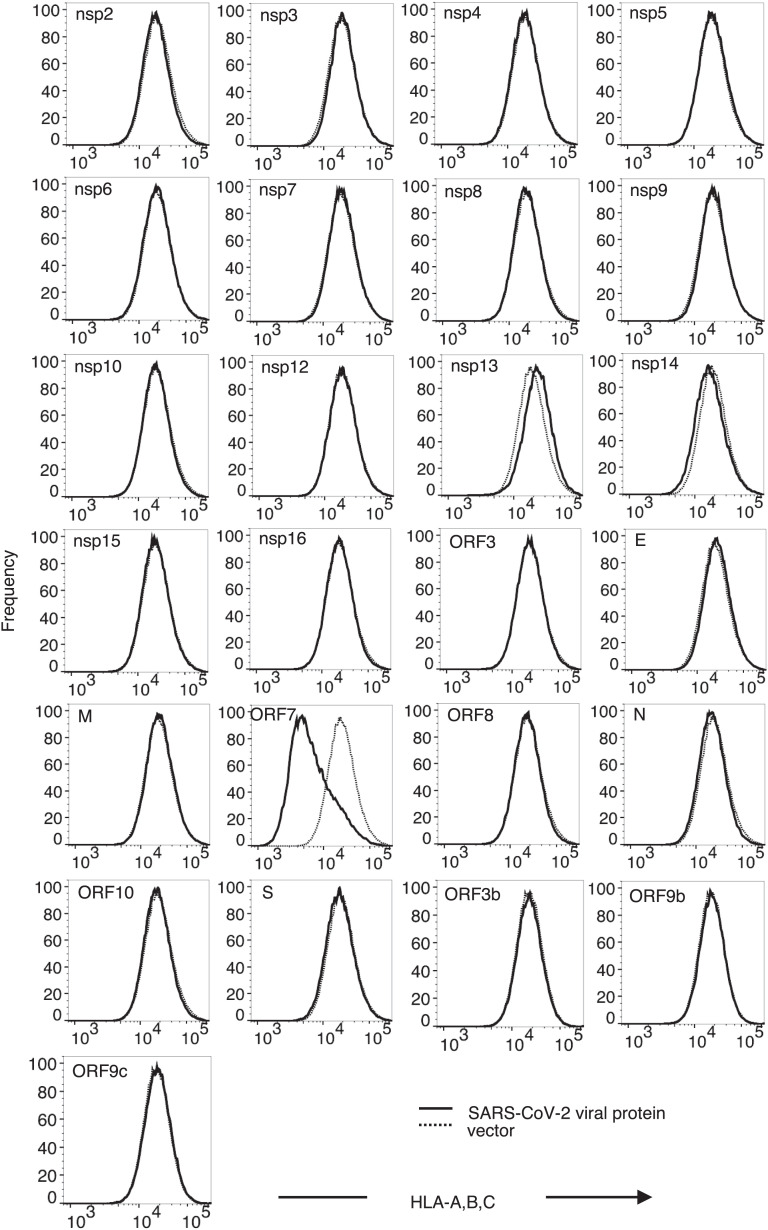
Effect of SARS-COV-2 ORFs on MHC-I cell surface levels in 293T cells. Human 293T cells transduced with SCRPSY-based lentiviral vector expressing individual SARS-CoV-2 viral proteins, at an MOI of 0.5 and cell surface MHC-I detected with a pan-HLA-ABC antibody (W6/32) and flow cytometry two days later. Cells transduced (TagRFP+ population) with viral protein expression vector (solid line) and empty lentiviral vector (dotted line) were gated and compared. Representative of three experiments.

To elucidate biological activities associated with individual SARS-CoV-2 viral proteins, we used an HIV-1-based lentiviral vector (pSCRPSY) (18) to express each SARS-CoV-2 viral open reading frame, as annotated in Wu et al. (13) and Gordon et al. (14). Two days after transduction of human 293T cells, we measured MHC-I surface levels by flow cytometry using a pan-HLA class I-reactive monoclonal antibody. Expression of ORF7a reduced MHC-I levels on the cell surface by approximately fivefold, whereas expression of other individual viral proteins [notably including ORF8 (16, 17)] had no effect on MHC-I surface levels (Fig. 1). We also examined the impact of SARS-CoV-2 ORFs on the expression of tetherin, a cell surface antiviral protein that traps enveloped virions from various virus families that bud through cell membranes. None of SARS-CoV-2 viral ORFs reduced the levels of tetherin stably expressed on the surface of 293T cells (*SI Appendix*, Fig. S1), underscoring the specificity of the effect of ORF7a on MHC-I. Of note, expression of two viral proteins (nsp1 and ORF6) was not accomplished in our screen, as lentiviral vectors encoding these proteins were low titer, in line with the previous findings that nsp1 suppresses host protein synthesis (19) and ORF6 interferes with nuclear transport machinery (14, 15).

**Fig. 2.**
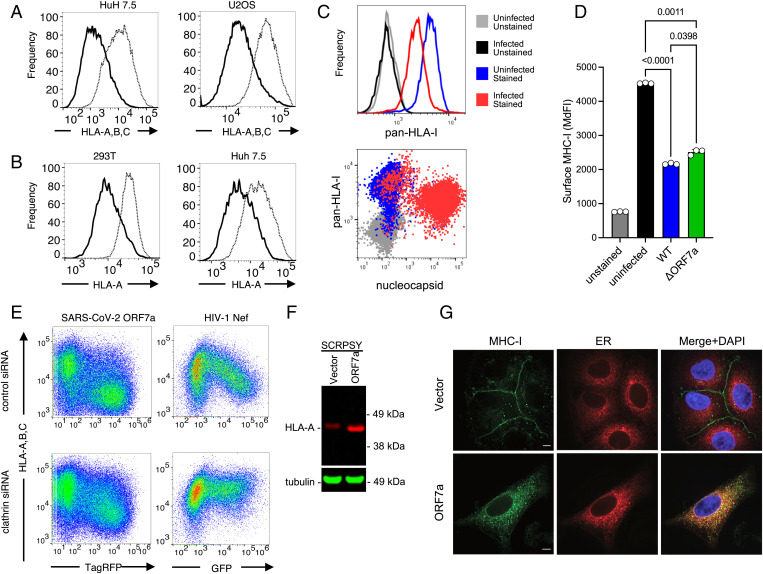
MHC-1 down-regulation by ORF7a. (*A*) HuH7.5 cells, U2OS cells, or 293T cells transduced with pSCRPSY-based lentiviral vector expressing SARS-CoV-2 ORF7a at an MOI of 0.5 and stained with a pan-HLA-ABC antibody (W6/32) or an HLA A-allele-specific antibody (YTH 862.2) followed by flow cytometry. (*B*) Cells transduced (TagRFP+ population) with the ORF7a expression vector (solid line) and empty lentiviral vector (dotted line) were gated and compared. Representative of three experiments. (*C*) A549/ACE2 cells stained with stained with a pan-HLA-ABC antibody (W6/32) and SARS-CoV-2 nucleocapsid antibody after infection with SARS-CoV-2_USA-WA1/2020_. (*D*) MHC-I surface levels (median fluorescent intensity [MdFI]) in A549/ACE2 cells stained with a pan-HLA-ABC antibody (W6/32) after infection with SARS-CoV-2_USA-WA1/2020_ (WT) or icSARS-CoV-2-mNG (ΔORF7). (*E*) Flow cytometric analysis of 293T cells with a pan-HLA-ABC antibody (W6/32) following transduction with a pSCRPSY-lentiviral vector expressing SARS-CoV-2 ORF7a (left panels) or a lentivirus vector expressing Nef and GFP (*Right Panels*), at an MOI of 0.5. Cells were transfected with control (*Upper Panels*) or clathrin HC-depleting (*Lower Panels*) siRNA prior to transduction. Representative of three experiments. (*F*) Western blot analysis of 293T cells 48 h following transduction with empty (vector) or SARS-CoV-2 ORF7a expressing SCRPSY with anti-HLA-A(red) and anti-Tubulin (green). (*G*) Immunofluorescent staining of A549 cells with anti HLA-A (green) following transduction with empty SCRPSY (vector) or ORF7a-expressing SCRPSY (ORF7a) and ER-GFP BacMam (red) a single optical section following deconvolution microscopy is shown.

ORF7a caused reduced MHC-I cell surface levels in other human cells such as Huh7.5 and U2OS (Fig. 2*A*) suggesting that its activity is not cell-type-specific. Because the recognition of MHC-I by the W6/32 antibody could be influenced by association between heavy chains (HC) and β2-microglobulin (β2M) (20, 21), we next confirmed that down-regulation occurred, as assessed with a different antibody, specific for the HLA-A HC (Fig. 2*B*). MHC-I surface levels were also depleted following SARS-CoV-2_USA-WA1/2020_ infection of A549/ACE2 cells, specifically in the infected nucleocapsid-positive subpopulation (Fig. 2 *C* and *D*). However, MHC-I down-regulation was largely maintained in cells infected with SARS-CoV-2 lacking ORF7a, suggesting the existence of additional, redundant means of MHC-I down-regulation (Fig. 2 *C* and *D*). We did not observe the previously reported MHC-I down-regulation induced by ORF8 expression, nor expression of other individual SARS-CoV-2 ORFs (Fig. 1), suggesting that additional mechanisms of MHC-I down-regulation require the concerted action of multiple SARS-CoV-2 proteins.

After loading with peptides in the endoplasmic reticulum (ER), MHC-I molecules are transported via the Golgi to the plasma membrane and are subsequently turned over by endocytosis (22, 23). To begin to elucidate how ORF7a might reduce cell surface MHC-I levels, we first depleted clathrin with small interfering RNA and found that clathrin knockdown, as reported previously (24), inhibited the down-regulation of MHC-I by Nef (Fig. 2*E*). Conversely, clathrin depletion had only a minor effect on MHC-I down-regulation by ORF7a, suggesting that ORF7a disturbs surface MHC-I levels in a distinct, clathrin-independent manner (Fig. 2*E*). Western blot analysis revealed no deficit in the total amount of MHC-I in cells expressing ORF7a. Indeed, when cells were transduced with the ORF7a lentivirus at sufficient multiplicity of infection (MOI) that most cells expressed ORF7a, the total amount of endogenous cellular MHC-I was increased, and displayed slightly accelerated migration, suggesting that ORF7a induces intracellular accumulation and altered posttranslational modification of MHC-I (Fig. 2*F*). Immunofluorescent staining of endogenous MHC-I in A549 cells showed that ORF7a profoundly altered the subcellular distribution of MHC-I molecules (Fig. 2*G*). Specifically, ORF7a induced a reduction in of MHC-I HC fluorescence that could be observed at the cell surface, consistent with the flow cytometric analysis (Fig. 2*A*), while intracellular MHC-I HC accumulated (Fig. 2*G*). The accumulated intracellular MHC-I HC was partly colocalized with a marker of the ER (Fig. 2*G*). The MHC-I component β2M was similarly redistributed to intracellular locations upon ORF7a expression and partly colocalized with ORF7a (*SI Appendix*, Fig. S2). We conclude that SARS-CoV-2 ORF7a blocks the ability of MHC-I to move through the secretory pathway to the cell surface.

### Determinants of Sarbecovirus ORF7a MHC-I Down-Regulation Activity.

**Fig. 3.**
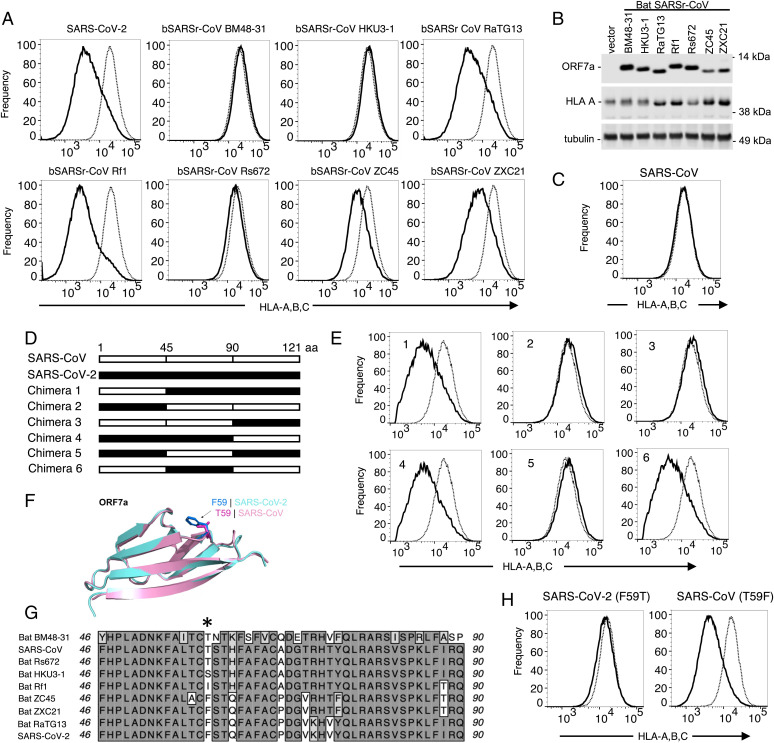
Determinants of MHC-I down-regulation by sarbecovirus ORF7a proteins. (*A*) Flow cytometric analysis of 293T cells with a pan-HLA-ABC antibody (W6/32) following transduction with a pSCRPSY-lentiviral vector expressing ORF7a proteins from SARS-CoV-2 bat SARSr coronaviruses BM48-31/BGR/2008 (YP_003858589.1), HKU3-1 (AAY88871.1), RaTG13 (QHR63305.1), Rf1/2004 (ABD75319.1), Rs_672/2006 (ACU31037.1), ZC45 (AVP78036.1), and ZXC21 (AVP78047.1). Cells transduced (TagRFP^+^ population) with the ORF7a expression vector (solid line) and empty lentiviral vector (dotted line) were gated and compared. Representative of three experiments. (*B*) Western blot analysis of 293T cell lysates following transduction with SCRPSY with no ORF7a (vector) or the ORF7a proteins from bat SARS-like coronaviruses as in (*A*). (*C*) Flow cytometric analysis of 293T cells with a pan-HLA-ABC antibody (W6/32) following transduction with a pSCRPSY-lentiviral vector expressing SARS-CoV. Cells transduced (TagRFP^+^ population) with the ORF7a expression vector (solid line) and empty lentiviral vector (dotted line) were gated and compared. (*D*) Schematic representation of chimeras generated between SARS-CoV and SARS-CoV-2 ORF7a proteins. (*E*) Flow cytometric analysis of 293T cells with a pan-HLA-ABC antibody (W6/32) following transduction with a pSCRPSY-lentiviral vector expressing chimeric ORF7a proteins shown in (*D*). Cells transduced (TagRFP^+^ population) with the ORF7a expression vector (solid line) and empty lentiviral vector (dotted line) were gated and compared. (*F*) Superimposed structures of the globular domains of SARS-CoV-2 and SARS-CoV ORF7a proteins indicating position of residue 59. (*G*) Amino acid alignment of SARS-CoV, SARS-CoV-2, and bat SARSr-CoV ORF7a proteins. Identical residues are shaded. Star indicates the amino acid at position 59 that were mutated in proteins used in (*H*). (*H*) Flow cytometric analysis of 293T cells with a pan-HLA-ABC antibody (W6/32) following transduction with a pSCRPSY-lentiviral vector expressing point mutant ORF7a proteins. Cells transduced (TagRFP^+^ population) with the ORF7a expression vector (solid line) and empty lentiviral vector (dotted line) were gated and compared.

We tested whether ORF7a proteins from a panel of bat SARS-related coronaviruses (SARSr-CoV), namely BM48-31, HKU3-1, Rf1, Rs672, ZC45, ZXC21, and RaTG13, shared the ability to reduce cell surface MHC-I levels (25). The RaTG13, Rf1, ZC45, and ZXC21 ORF7a proteins, but not those from BM48-31, HKU3-1, and Rs672 ORF7, reduced MHC-I surface levels (Fig. 3*A*). Western blot and immunofluorescence analyses showed that the sarbecovirus ORF7a proteins were expressed in transduced cells at a similar level and those that induced surface down-regulation also induced intracellular accumulation and altered migration of MHC-I (Fig. 3*B* and *SI Appendix*, Fig. S3).

Notably, the ORF7a protein from SARS-CoV was among those that did not affect MHC-I surface levels (Fig. 3*C*), despite encoding 85% identical amino acids to SARS-CoV-2 ORF7a. Moreover, unlike SARS-CoV-2 ORF7a, and the active bat SARSr-CoV ORF7a proteins, the SARS-CoV ORF7a protein showed little tendency to colocalize with MHC-I (*SI Appendix*, Fig. S4).

To map the determinants of the differential ability to modulate cell surface MHC-I levels, we constructed six chimeric SARS-CoV-2/SARS-CoV ORF7a expression vectors (Fig. 3*D*). Chimeric ORF7a proteins containing the central region (residues 46–90) from SARS-CoV-2 all down-regulated MHC-I from the cell surface while those encoding the equivalent region from SARS-CoV did not (Fig. 3*E*). Among the sarbecovirus ORF7a proteins, the presence of hydrophobic residues (F or I) at position 59 within this region that is predicted to be surface exposed (Fig. 3*F*) correlated with the ability to down-regulate MHC-I, while the presence of a polar residue (T or S) correlated with inactivity (Fig. 3*G*). Thus, to test the importance of the amino acid at position 59, we introduced an F59T substitution into SARS-CoV-2 ORF7a and found this substitution abolished MHC-I downregulating activity, while the reciprocal substitution in SARS-CoV ORF7a (T59F) substitution led to acquisition of MHC-I downregulating activity (Fig. 3*H*). We conclude that residue 59, a variable position among sarbecovirus ORF7a proteins, is a critical determinant of their ability to modulate cell surface MHC-I levels. Notably, neither SARS-CoV-2, SARS-CoV, nor any of the residue 59 mutants thereof could down-regulate MHC-I (H-2L^d^) in murine NIH 3T3 cells, suggesting that either interactions between ORF7a and target or effector proteins are species-specific (*SI Appendix*, Fig. S5).

### Interaction between MHC-I and ORF7a.

**Fig. 4.**
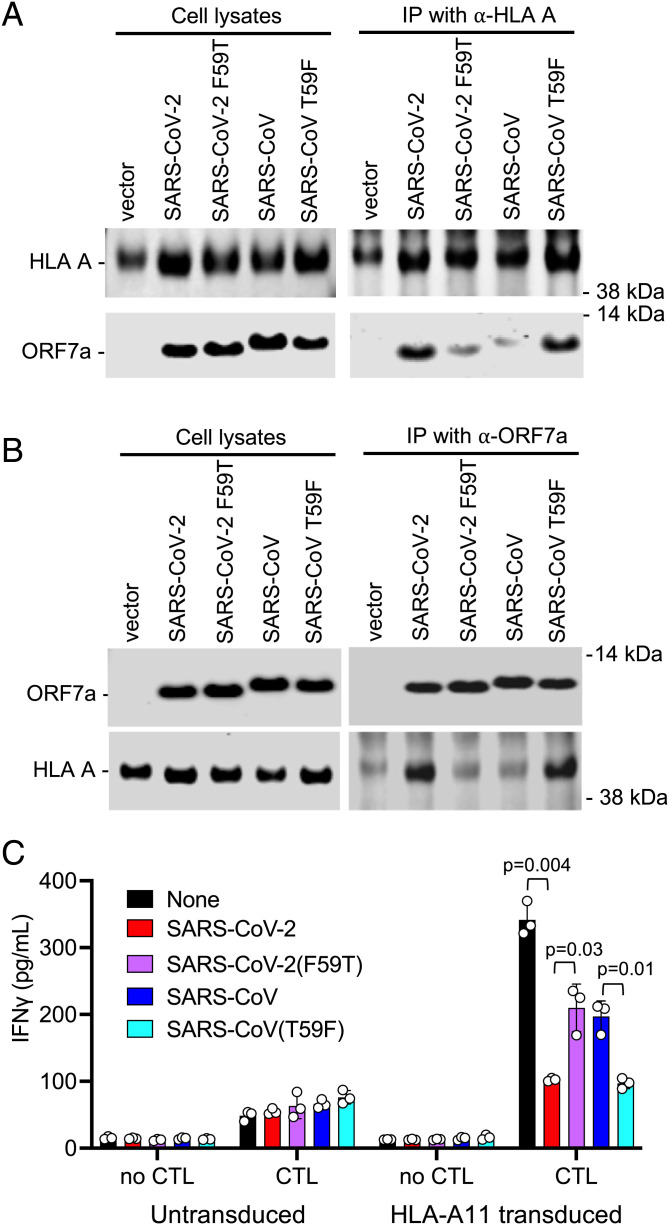
ORF7a physically associated with MHC-I and inhibits antigen presentation. (*A* and *B*) Western blot analysis of cell lysates and immunoprecipitates (IP) from 293T cells transduced with SCRPSY expressing no ORF7a (vector) or the indicated wild-type (WT) or mutant ORF7a proteins. Immunoprecipitation was carried out with a mouse HLA class I ABC monoclonal antibody (66013-1-Ig) and precipitated ORF7a, and MHC-I proteins were detected with rabbit anti-ORF7a antibody and an HLA A-specific antibody (EP1395Y), respectively (*A*). Alternatively, immunoprecipitation was carried out with mouse anti-SARS-CoV ORF7a monoclonal antibody (3C9), and precipitated ORF7a and MHC-I proteins were detected with rabbit anti-ORF7a and an HLA A-specific antibody (EP1395Y) (*B*). Representative of three experiments. (*C*) Levels of IFN-γ in supernatants following 16 h of incubation of the AK11 CD8^+^ T cell clone with 293T target cells that were either untransduced or transduced with an HLA-A11 expression vector, followed by an ORF7a expressing (or control) SCRPSY vector, and then transfected with an HIV-1 Gag expression plasmid. *P* values are calculated using the *t* test with Welch’s correction.

To test whether ORF7a proteins could physically associate with MHC-I, we immunoprecipitated the endogenously expressed MHC-I HC, in ORF7a-expressing cells. The SARS-CoV-2 ORF7a protein could be coimmunoprecipitated from transduced cells with an anti-HLA-A antibody, and the amount of coimmunoprecipitated ORF7a, but not the total level of ORF7a protein, was reduced to background levels by the functionally inactivating F59T substitution in SARS-CoV-2 ORF7a (Fig. 4*A* and *SI Appendix*, Fig. S6*A*). Conversely, SARS-CoV ORF7 was poorly coimmunoprecipitated by the anti-HLA-A antibody, but the T59F gain of function substitution increased the amount of coimmunoprecipitated ORF7a (Fig. 4*A* and *SI Appendix*, Fig. S6*A*). In reciprocal immunoprecipitation experiments, HLA-A could be immunoprecipitated by an ORF7a antibody from cells expressing wild-type SARS-CoV-2 ORF7a or SARS-CoV ORF7a(T59F) mutant (Fig. 4*B* and *SI Appendix*, Fig. S6*B*). Conversely, expression of the SARS-CoV-2 ORF7a(F59T) mutant or wild-type SARS-CoV ORF7a resulted in less efficient coprecipitation of the HLA-A protein (Fig. 4*B* and *SI Appendix*, Fig. S6*B*). We conclude that ORF7a associates with MHC-I HC and that single amino acid substitutions in ORF7a that confer or ablate MHC-I downregulating activity simultaneously confer or ablate the ability of ORF7a to coimmunoprecipitate with MHC-I.

### ORF7a Inhibits MHC-I Antigen Presentation to a CD8^+^ T Cell Clone.

To determine whether SARS-CoV-2 ORF7a could inhibit antigen presentation, we engineered 293T cells to express HLA-A11 and then expressed an HIV-1 Gag protein and an ORF7a protein therein. We used these HIV-1 Gag-expressing cells as “targets” for an HLA-A11 restricted CD8^+^ T cell clone that recognizes the HIV-1 Gag peptide ACQGVGGPSHK (AK11) and measured the ability of the CD8^+^ T cell clone to respond to the Gag-expressing target cells by secreting interferon (IFN)-γ). The AK11 CD8^+^ T cell clone indeed responded to Gag-expressing 293T cells by secreting IFN-γ, and this response was inhibited, almost to background levels, when the 293T target cells also expressed SARS-CoV-2 ORF7a (Fig. 4*C*). The loss of function (F59T) SARS-CoV-2 ORF7a mutant and the wild-type SARS-CoV ORF7a proteins were less potent in reducing AK11 CD8^+^ T cell clone responsiveness. Conversely, the SARS-CoV gain of function (T59F) mutant reduced the IFN-γ response as potently as the WT SARS-CoV-2 ORF7a protein (Fig. 4*C*).

### Disruption of the MHC-I Peptide Loading Complex and ER Retention Induced by ORF7a.

**Fig. 5.**
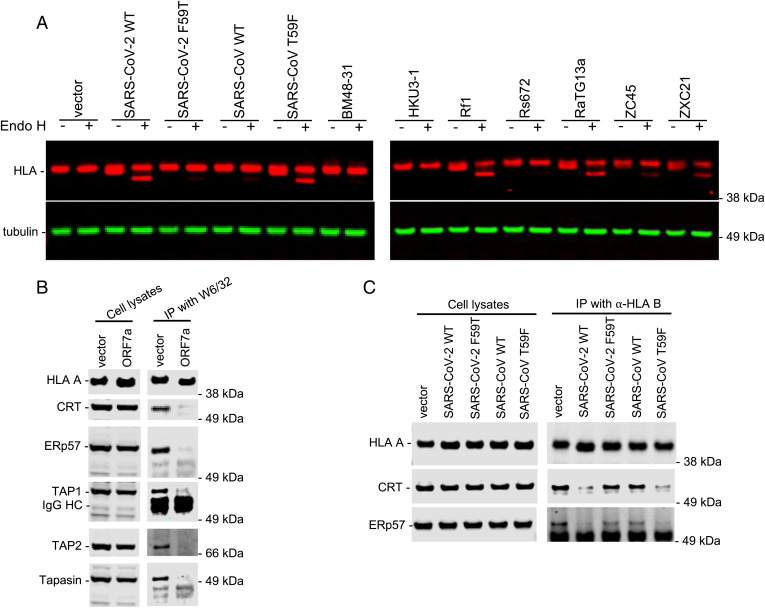
ER retention and disruption of the MHC-1 peptide loading complex assembly by ORF7a. (*A*) Western blot analysis of cell lysates of A549 cells transduced with SCRPSY expressing no ORF7a (vector) or the ORF7a proteins from SARS-CoV-2, SARS-CoV (or mutants thereof), or ORF7a proteins from the indicated bat SARSr-CoVs at an MOI of 0.5. Cell lysates were untreated or treated with endoglycosidase H, as indicated prior to analysis MHC-I proteins, and tubulin were detected with mouse anti-HLA-A, HLA-B, and HLA-C antibody (Abcam ab70328) and anti-tubulin antibody, respectively. (*B*) Western blot analysis of cell lysates and immunoprecipitates (IP) from 293T cells transduced with SCRPSY expressing no ORF7a (vector) or the SARS-CoV-2 ORF7a protein. Immunoprecipitation was carried out with a pan-HLA-ABC antibody (W6/32). Immunoprecipitated proteins were detected with rabbit anti-calreticulin antibody (CRT), anti-ERp57 antibody, anti-TAP1 antibody, anti-TAP2 antibody, and anti-Tapasin antibody. Representative of three experiments. (*C*) Western blot analysis of cell lysates and IP from 293T cells transduced with SCRPSY expressing no ORF7a (vector) or the indicated WT and mutant ORF7a proteins. Immunoprecipitation carried out with a rabbit HLA-B specific antibody (PA5-35345). Immunoprecipitated proteins were detected with mouse anti-Calreticulin antibody and anti-ERp57 antibody, respectively. Representative of three experiments.

We next attempted to determine more precisely how ORF7a interferes with MHC-I transport through the secretory pathway and with antigen presentation at the cell surface. As MHC-I moves through the Golgi, a single *N*-linked glycan on the HC is remodeled from an initially endoglycosidase H (Endo H)-sensitive high mannose form to a complex Endo H-resistant form (22, 26, 27). Western blot analysis of Endo H-treated cell lysates from control A549 cells indicated that at steady state, the majority of MHC-I HC glycan was Endo H-resistant, while in a cell population in which the cells expressed ORF7a proteins, approximately 50% of the HLA-A HC was Endo H-sensitive (Fig. 5*A* and *SI Appendix*, Fig. S7), consistent with the intracellular accumulation of MHC-I in SARS-CoV-2 ORF7a-expressing cells (Fig. 2*C* and *SI Appendix*, Fig. S2 and Fig. S4). The inactive SARS-CoV-2(F59T) and wild-type SARS-CoV proteins did not induce the appearance of the Endo H-sensitive species while the SARS-CoV(T59F) gain of function mutant had acquired this activity. Moreover, the abilities of members of the panel of bat SARSr-CoV proteins to deplete MHC-I proteins from the cell surface correlated with their abilities to induce the accumulation of Endo H-sensitive MHC-I HC species (Fig. 5*A* and *SI Appendix*, Fig. S7). As controls, we determined the ability of wild-type and mutant SARS-CoV-2 and SARS-CoV ORF7a proteins to affect the Endo H sensitivity of two other glycoproteins (CD44 and CDCP1) that are not associated with MHC-I. The ORF7a proteins did not affect the Endo-H sensitivity/resistance of either protein (*SI Appendix*, Fig. S8). We conclude that the sarbecovirus ORF7a proteins which cause depletion of cell surface MHC-I do so by specifically inducing retention of MHC-I in premedial Golgi compartment(s), and not through toxicity or nonspecific effects on the secretory pathway.

For antigen presentation, peptides are loaded into MHC-I in the ER through an interaction of MHC-I with the peptide loading complex (PLC). The PLC includes the transporter associated with antigen processing (TAP) proteins, TAP1 and TAP2, as well as chaperones/cofactors Tapasin, ERp57, and calreticulin that each bind directly or indirectly to the MHC-HC/β2 microglobulin complex in the ER lumen (9). Western blot analysis of proteins that coimmunoprecipitated with endogenous MHC-I revealed that expression of SARS-CoV-2 ORF7a blocked the coimmunoprecipitation of each the aforementioned MHC-I associated proteins with MHC-I HC without affecting the steady-state levels of these proteins (Fig. 5*B* and *SI Appendix*, Fig. S9*A*). To confirm the specificity of these effects, we employed the SARS-CoV-2 loss-of-function (F59T) and SARS-CoV gain-of-function (T59F) mutants. In similar coimmunoprecipitation assays in which endogenous HLA-B was immunoprecipitated, the coimmunoprecipitation of calreticulin and ERp57 was inhibited by the active WT SARS-CoV-2 and SARS-CoV(T59F) ORF7a proteins but not by the inactive SARS-CoV-2(F59T) and WT SARS-CoV ORF7a proteins (Fig. 5*C* and *SI Appendix*, Fig. S9*B*). We conclude that active ORF7a proteins disrupt the assembly of the MHC-I PLC in the ER and prevent the movement of peptide loaded MHC-I complexes to the cell surface for antigen presentation.

## Discussion

Inhibition of antigen presentation through the down-regulation of MHC-I peptide complexes is a long-recognized means by which certain viruses mitigate the inhibitory action of immune responses on their replication (10). MHC-I down-regulation is frequently associated with viruses that cause persistent or chronic infection, or viruses with large DNA genomes and many immunomodulatory genes (11). Such down-regulation is less frequently associated with RNA viruses or those that cause acute, rapidly resolving infections (11). Nevertheless, by reducing the density of viral peptides that are displayed on the surface of infected cells, viruses that cause acute infections, such as SARS-CoV-2, could gain a competitive advantage through MHC-I down-regulation. This advantage would be most pronounced in contexts where preexisting T cell responses could limit virus replication, such as reinfection, and might also extend to limiting the protective effects of preexisting T cell responses to epitopes that are conserved in distinct coronaviruses (28). Moreover, while SARS-CoV-2 is typically viewed as an acute respiratory infection, it is increasingly recognized that SARS-CoV-2 infection can persist for months in certain anatomical sites (e.g., the gut) or in individuals with suboptimal immune responses (29–31). Moreover, the natural history of sarbecovirus infection in nonhuman species such as bats, and the degree to which it is a persistent infection, is poorly documented. Thus, there are several reasons to think that MHC-I down-regulation might be an evolved property in sarbecoviruses.

Some recent studies have suggested that SARS-CoV-2 ORF8 protein induces MHC-I down-regulation (16, 17). We did not observe such an effect. Nevertheless, an ORF7a-deleted SARS-CoV-2 induced MHC-I down-regulation almost as efficiently as did an ORF7a-intact virus. This finding suggests that additional redundant mechanisms are employed by SARS-CoV-2 to deplete surface MHC-I and reduce visibility to responding CD8^+^ T cells. For example, a recent study has suggested that SARS-CoV-2 ORF6 inhibits transcriptional up-regulation of MHC-I in response to infection (32).

The variation among the ability of bat SARSr-CoV ORF7a proteins to reduce surface MHC-I levels is intriguing. It is unclear whether MHC-I down-regulation is a “natural” function of ORF7a in bat hosts or whether its ability to down-regulate MHC-I in humans is fortuitous. While the precise details of mechanisms by which ORF7a cause PLC disruption and ER retention of MHC-I warrant further investigation, ORF7a itself is a ER-resident transmembrane protein consisting of a signal sequence, a globular luminal domain, a transmembrane domain and a cytosolic ER retention “KRK” signal. The anatomy of ORF7a appears therefore to be ideally suited to interact with proteins in the secretory pathway and cause ER retention. The structure of the luminal domains SARS-CoV and SARS-CoV-2 ORF7a proteins is nearly identical, resembles an immunoglobulin domain, and position 59 is a surface exposed position. Variation at this critical determinant of MHC-I down-regulation could reflect genetic conflict with variable MHC-I proteins in animal hosts, and a lack of historical adaptation to humans. We found that ORF7a does not down-regulate MHC-I in murine cells, but the extent to which ORF7a exhibits this activity in other mammalian species, including natural hosts is unclear. While it is counterintuitive that the MHC-I downregulating activity of ORF7a is not conserved across the SARS-related coronaviruses, it is plausible (even likely) that the ORF7a MHC-I interaction might be a site of genetic conflict in natural hosts. Thus, it might be expected that loss versus retention of this activity in nonnatural hosts might be sporadic. In any case, the differential ability of members of this pandemic threat virus subgenus to manipulate human MHC-I molecules could impact their ability to evade CD8^+^ T cell immunity elicited by prior sarbecovirus infection or vaccination, with substantial potential to affect human health.

## Materials and Methods

### Antibodies.

Primary antibodies, including anti-ORF7a, anti-pan HLA ABC, HLA allele-specific antibodies, and peptide-loading complex antibodies, are listed and described in the *SI Appendix*, Table S1. Secondary antibodies included goat anti-rabbit and goat anti-mouse from Novus conjugated to DyLight 650 or Janelia Fluor 646 and goat anti-mouse from ThermoFisher Scientific conjugated to Alexa Flour 488 for immunofluorescence, or IRDye 800CW, or IRDye 680 (LI-COR Biosciences) for Western blot analysis.

### Plasmid Construction.

All SARS-CoV-2 viral proteins, including nonstructural proteins (NSPs), open-reading frames (ORFs), and structural proteins (E, M, N and S) annotated in the viral genome [GenBank: MN985325 (13, 14)] were human codon-optimized using GenSmart Codon Optimization and synthesized by IDT DNA Technologies as gBlocks. ORF7a genes from SARS-CoV and the various bat SARSr-CoVs were synthesized based on their amino acid sequences, which included SARS coronavirus Tor2 (GenBank: AAP41043.1), bat coronavirus BM48-31/BGR/2008 (YP_003858589.1), bat SARS coronavirus HKU3-1 (AAY88871.1), bat SARS CoV Rf1/2004 (ABD75319.1), bat SARS coronavirus Rs_672/2006 (ACU31037.1), bat coronavirus RaTG13 (QHR63305.1), bat SARS-like coronavirus ZC45 (AVP78036.1), and bat SARS-like coronavirus ZXC21 (AVP78047.1). The synthesized nucleotide sequences are listed in the *SI Appendix*, Dataset S2. For each gene fragment, XhoI and NotI sites were added at 5′ end and 3′ end, respectively, and a typical Kozak sequence (GCCACC) was inserted before each start codon (ATG). The synthetic genes were digested with XhoI and NotI and inserted into an HIV-1-based lentiviral expression vector (pSCRPSY) which also expresses a puromycin-resistance cassette and TagRFP.

To make the SARS-CoV (T59F) or SARS-CoV-2 ORF7a (F59T) expression vectors, overlap-extension PCR amplification were performed with primers that incorporated the corresponding nucleotide substitutions using corresponding wild-type plasmids as template, respectively. After digestion with XhoI and NotI, the purified PCR products were then inserted into the pSCRPSY expression vector. HLA A11 gene fragment was PCR amplified and, after digestion with XhoI and NotI, subcloned into a retroviral vector (LMNi-bsd).

### Cell Lines.

The human embryonic kidney HEK-293T cell line, human hepatoma-derived HuH-7.5 cell line, and human alveolar basal epithelial A549 cell line were maintained in DMEM supplemented with 10% fetal bovine serum (Sigma F8067) and gentamicin (Gibco). Human bone osteosarcoma epithelial cells (U2OS, ATCC HTB-96) were grown in McCoy's 5a Medium Modified (ATCC 30–2007)/10% FCS/gentamicin. All cell lines used in this study were monitored by SYBR Green real-time PCR RT assay periodically to ensure the absence of retroviral contamination and were stained with DAPI to test for mycoplasma contamination.

### Assessment of MHC-I Down-Regulation by ORF7a.

To assess HLA down-regulation by SARS-CoV viral proteins, viral stocks were generated by transfecting 5 μg of pSCRPSY-based expression plasmids encoding either no SARS-CoV viral protein (empty vector) or individual SARS-CoV open reading frame, 5 μg of an HIV-1 Gag-Pol expression plasmid (pCRV1/GagPol), and 1 μg of VSV-G expression plasmid in 293T cells in 10-cm dishes using polyethylenimine (PolySciences). Virus-containing supernatant was collected and filtered (0.2 μm) 2 d later. The viral stocks were then used to inoculate10^5^ target cells (human 293T, U2OS, or HuH7.5 or mouse NIH 3T3 cells engineered to express human CycT1 to enable efficient expression from the pSCRPSY HIV-1 vector) in 12-well plates at an MOI of 0.5. At 48 h posttransduction, cells were detached from plates with 5 mM EDTA in PBS and stained for cell surface MHC-I expression with anti-HLA-A, HLA-B, HLA-C antibody conjugated to AF647 (W6/32, Biolegend) for human cells or anti-mouse H-2L^d^ conjugated to Alexa Fluor488 (Clone 30–5-7S) (Cedarlane, Cat CL9011AF4) for mouse cells. In some experiments, cell surface HLA-A level was determined with antibody against the HLA-A allele (YTH862.2), followed by goat anti-rat Alexa Fluro 488. The same procedure was done to quantify tetherin down-regulation, except that 293T cells stably expressing HA-tagged tetherin were used as target cells, and cells were stained with anti-HA.11 antibody (Biolegend). Flow cytometric analysis was performed using Attune NxT Acoustic Focusing Cytometer (ThermoFisher Scientific).

To assess the effect of clathrin knockdown on MHC-I down-regulation by SARS-CoV-2 ORF7a or HIV Nef, 293T cells were seeded 1 d prior to transfection with clathrin small interfering RNA (siRNA) (Horizon Discovery, Dharmacon) using Invitrogen Lipofectamine RNAiMax (ThermoFisher Scientific). One day later, the cells were transduced with lentivirus virus stocks expressing SARS-CoV-2 ORF7a or HIV Nef and harvested for fluorescence-activated cell sorter analysis 48 h later.

### Assessment of MHC-I Down-Regulation after Live SARS-CoV-2 Infection.

Viral stocks of USA-WA1/2020 (BEI Resources) and icSARS-CoV-2-mNG [UTMB WRCEVA (33)] were generated by expanding virus on Vero-E6 cells (BEI Resources) and determining viral titers by plaque assay on Vero-E6 cells. A549-ACE2 cells (ATCC) were plated at 1 × 10^6^ cells per well in a 6-well plate in DMEM (Corning) supplemented with Hepes (Corning), penicillin/streptomycin (Corning), GlutaMAX (Thermo Fisher Scientific), and 10% fetal bovine serum (FBS) (Sigma) the day before infection. The cells were then infected at MOI 0.1 for 1 h at 37 °C, after which the overlying media was replaced with DMEM supplemented with Hepes, penicillin/streptomycin, GlutaMAX, and 2% FBS. After 48 h, cells were washed with PBS (Corning) and harvested using TrypLE (Life Technologies) before staining with Live/Dead Blue dead cell stain (Invitrogen) and a BV510-conjugated pan-HLA class I antibody (clone W6/32, BioLegend). Cells were fixed with 4% paraformaldehyde (Santa Cruz Biotechnology) and subsequently permeabilized with Perm/Wash Buffer (BD Biosciences). Intracellular staining was performed in Perm/Wash Buffer with an AF647-conjugated pan-HLA class I antibody (clone W6/32, BioLegend) and a SARS-CoV-2 nucleocapsid antibody (clone A20087H, mouse IgG2b isotype, BioLegend) followed by a secondary antibody stain with PE-Cy7-conjugated anti-mouse IgG2b (BioLegend). Fixed and stained cells were then washed and prepared for flow cytometry. All live virus experiments performed in the Ragon Institute Biosafety Level 3 (BSL3) laboratory following protocols approved by the Mass General Brigham Institutional Biosafety Committee. Flow cytometry was performed on a BD Symphony (BD Biosciences, San Jose, CA) and analyses were performed using FlowJo v10.7.1 and GraphPad Prism 9 software.

### Immunoprecipitation.

HEK-293T cells were seeded at 2 × 10^5^ in 6-well plates and, on the next day, were inoculated with ORF7a-expressing SCRPSY lentivirus stocks. At 40 h postinfection, cells were detached by trypsinization and treated for 20 min with 10 mM methyl methanethiosulfonate (Thermo Scientific) in PBS on ice. Cells were then lysed with ice-cold lysis buffer (25 mM Tris, pH 7.4, 150 mM NaCl, 0.4% Nonidet P-40 (Sigma), 5% glycerol, supplemented with 1× complete protease inhibitor (Roche). After lysis on ice for 20 min, followed by centrifugation at 10,000 rpm for 10 min at 4 °C, clarified lysates were mixed with 2 µg mouse anti-ORF7a monoclonal antibody (Genetex), mouse anti-HLA-A (Proteintech 66013-1-Ig), rabbit anti-HLA-B (ThermoFisher Scientific PA5-35345), or mouse anti-HLA-A, B, C (W6/32) and rotated with 30 µL pre-equilibrated Protein G Sepharose 4 Fast Flow resin (GE healthcare) for 2 h at 4 °C. The resin was then washed 3 times with PBS, and the bound proteins were eluted with SDS-PAGE sample buffer and analyzed by Western blotting.

### Western Blot Analysis.

Cell lysates and immunoprecipitates were separated on NuPage Novex 4–12% Bis-Tris Mini Gels (Invitrogen), and NuPAGE MES SDS running buffer (Invitrogen, NP0002) was used. Proteins were blotted onto nitrocellulose membranes. Thereafter, the blots were probed with primary antibodies and followed by secondary antibodies conjugated to IRDye 800CW or IRDye 680. Fluorescent signals were detected using an Odyssey scanner (LI-COR Biosciences).

To measure the sensitivity of MHC-I to Endo H treatment, A549 cells were transduced with lentivirus stocks expressing ORF7a proteins from SARS-CoV, SARS-CoV-2, or bat SARSr CoVs at an MOI of 0.5. After 40 h, cells were treated with puromycin to remove untransduced cells and, after additional 20 h, cells were harvested and treated with Endo H (New England Biolabs) for 60 min. The whole cell lysates were loaded on SDS-PAGE for Western blot analysis.

### HIV-1 Gag-Specific T Cell Clones.

CD8^+^ T cell clones specific for the HLA-A11-restricted Gag epitope ACQGVGGPGHK (AK11) were isolated and expanded using a previously described protocol (34). Briefly, peripheral blood mononuclear cells (PBMCs) from an HIV-infected individual were thawed, washed in warm X-VIVO-15, and resuspended at a concentration of 1 × 10^7^ cells/mL PBMCs were stimulated for 3 h with 10 μg/mL of Gag AK11 peptide and T cells producing IFN-γ in response were enriched using the IFN-γ Secretion Detection and Enrichment Kit (130-054-201; Miltenyi Biotec) in accordance with the manufacturer’s instructions. Enriched T cells were plated at a series of dilutions in 96-well plates with irradiated feeder medium (RPMI 1640 supplemented with 10% FBS, l-glutamine, and PenStrep [R-10]) with 1 × 10^6^ cells/mL 5000 rad irradiated PBMC + 50 U/mL interleukin (IL)-2 + 10 ng/mL IL-15 (both from NCI BRB Preclinical Biologics Repository) + 0.1 μg/mL each of anti-CD3 (Ultra-LEAF purified anti-human CD3 antibody clone OKT3; BioLegend) and anti-CD28 (Ultra-LEAF purified Anti-human CD28 antibody clone 28.2; BioLegend), 37 °C 5% CO_2_. One month later, wells were selected from the most dilute plate that showed growth (<1/3 of wells containing cell populations) and each was screened for specificity to Gag-AK11 peptide pool by CD107a staining and flow cytometry. Specific clones were expanded through biweekly stimulations with irradiated feeder medium. Clone specificity was further confirmed by peptide stimulation and CD107a staining on the day before performing T cell antigen recognition assays.

### T Cell Antigen Recognition Assay.

To measure antigen presentation, 293T cells were transduced with a retrovirus vector (LMNi-bsd) expressing HLA A11 or an empty vector control. The cells were subsequently transduced with empty SCRPSY or SCRPSY expressing an ORF7a protein at an MOI of 1. After selection in puromycin for 24 h, cells were transfected with an HIV-1 Gag expression plasmid. these “target” cells were washed to remove the selection media, plated in 96-well plates and then cocultured with a Gag-AK11-specific CD8^+^ T cell clone in RPMI medium 1640 supplemented with 10% FBS, 2 mM l-glutamine, 100 units/mL penicillin, 100 μg/mL streptomycin, and 50 units/mL IL-2 (NCI BRB Preclinical Biologics Repository) at 37 °C 5% CO_2_. After 16 h, cells were pelleted, and supernatants were harvested for IFN-γ ELISA. Cells were then washed in 2% FBS phosphate-buffered saline + 2 mM EDTA and surface stained with fluorochrome-conjugated antibodies to CD3-Brilliant Violet 785 clone OKT3, CD8-BV605 clone SK1, MHC-I- PacBlue clone W6/32, (all from BioLegend), CD4-APC R700 clone RPA-T4, (BD), Gag KC57-FITC (Beckman Coulter), and Fixable Aqua Viability Dye (Invitrogen). Counting beads were also added. Samples were analyzed by flow cytometry, and data analysis was performed with FlowJo (version 10) software.

### IFN-γ ELISA.

Levels of IFN-γ in supernatants from T cell clone recognition assays were measured by ELISA using the ELISA MAX Deluxe Set Human IFN-g kit (Biolegend) following the manufacturer’s instructions. Concentrations were determined by interpolating from a standard curve.

### Deconvolution Microscopy.

Five thousand A549 cells were seeded onto gelatin (Millipore ES-0060B) coated 8-chamber #1.5 borosilicate glass bottom slides (LabTek 155409) and transduced with SCRPSY lentiviruses expressing ORF7a or a mutant thereof at an MOI of 1. For some wells, 2 µL of CellLight ER-GFP BacMam 2.0 (ThermoFisher C10590) was added at the time of infection. The cells were fixed at 48 h.p.i. with 4% formaldehyde (ThermoFisher 28908) in PBS (Thermo AM9624) for 15 min at RT. Cells were rinsed with PBS and blocked with 10% goat serum (Sigma G9023-10ML) in PBS for 20 min. Following 10 min permeabilization at room temperature with 0.2% IGEPAL CA-630 (Sigma I3021), 10% goat serum in PBS, cells were probed for 1 h at RT with primary antibodies in 0.1% Tween20 (Fisher BP337-500), 10% goat serum in PBS as indicated: HLA-A (Sigma H1650-100STS, 0.4 µg/mL), beta-2 microglobulin (Novus NBP2-44471, 0.4 µg/mL), or ORF7a (Bioworld NCP0011, 2 µg/mL). After three rinses with 0.1% Tween20 (Fisher BP337-500) in PBS, cells were probed with secondary antibodies for 45 min at RT in 0.1% Tween20, 10% goat serum, Hoechst 33258 (Abcam ab145596, 1 µg/mL) in PBS as indicated: goat anti-rabbit (Novus NBP1-72732C, 1.4 µg/mL), goat anti-mouse (Novus NBP1-72739JF646, 1.4 µg/mL or ThermoFisher Scientific A11029 4 µg/mL). Cells were rinsed twice with 0.1% Tween20 in PBS and twice with PBS and imaged by deconvolution microscopy (DeltaVision OMX SR imaging system). Image analysis was done using ImageJ (Version 2.0.0-rc-59/1.51w).

## Supplementary Material

Supplementary File

Supplementary File

## Data Availability

All study data are included in the article and/or *SI Appendix*.
