# Supplementary material for: Inhibition of major histocompatibility complex-I antigen presentation by sarbecovirus ORF7a proteins
Source: Proc Natl Acad Sci U S A. 2022 Sep 22;119(41):e2209042119. doi: 10.1073/pnas.2209042119 (PMC9573092; doi:10.1073/pnas.2209042119)
Supplement: Supplementary File [file pnas.2209042119.sapp.pdf]

PNAS

Supporting Information for

Inhibition of major histocompatibility complex-I antigen presentation by sarbecovirus ORF7a proteins

Fengwen Zhang, Trinity Zang, Eva M. Stevenson, Xiao Lei, Dennis C. Copertino, Talia M. Mota, Julie Boucau, Wilfredo F. Garcia-Beltran, R. Brad Jones, Paul D. Bieniasz

corresponding author: Paul D. Bieniasz

Email:pbieniasz@rockefeller.edu

This PDF file includes:

Figures S1 to S9
Tables S1
Legends for Datasets S1
SI References

Other supporting materials for this manuscript include the following:

Dataset S1

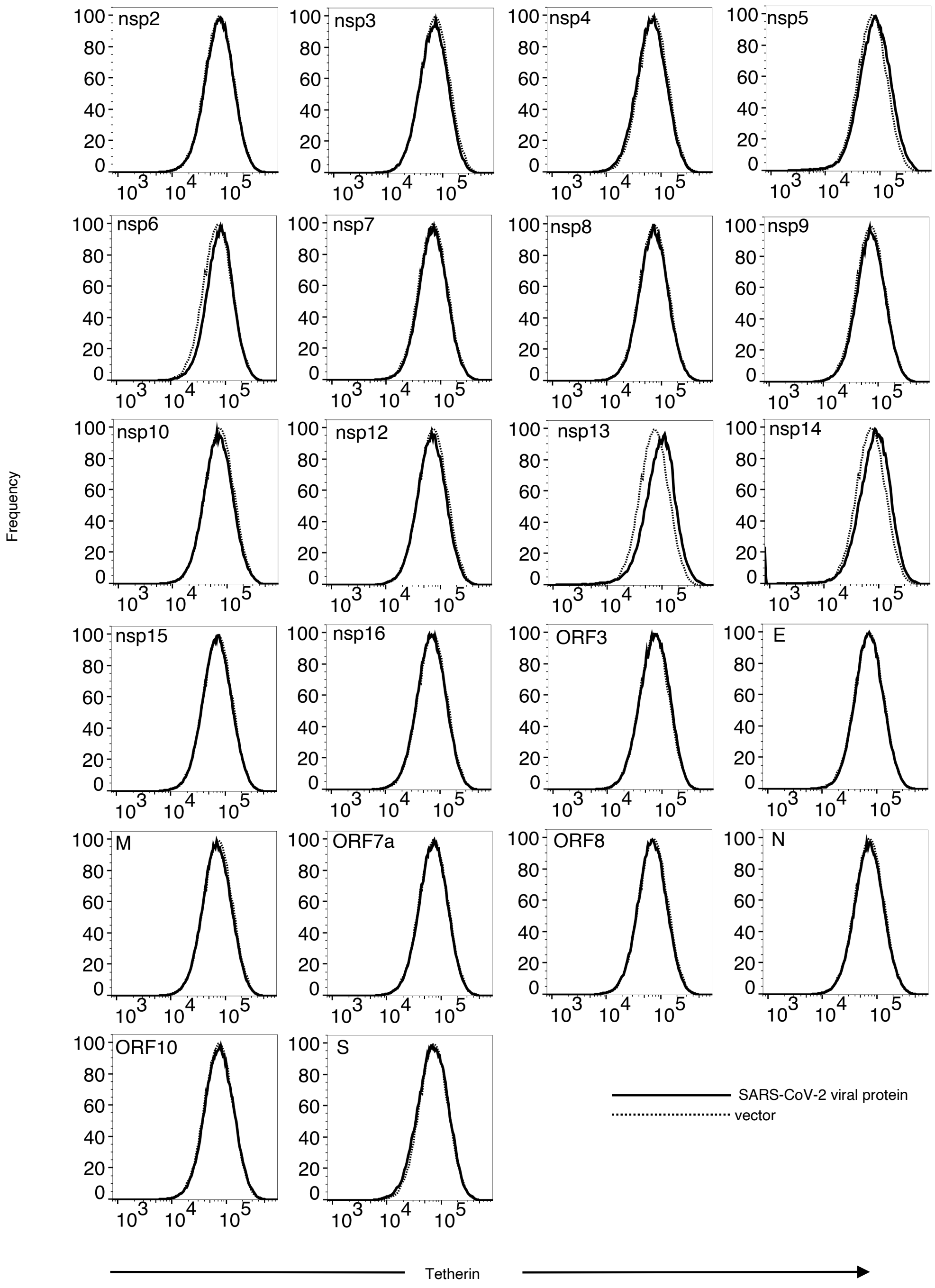

Figure S1 No effect of SARS-CoV-2 proteins on cell surface levels of Tetherin.
Human 293T cells transduced with pSCRPSY-based lentiviral vector expressing individual SARS-CoV-2 viral proteins, at an MOI of 0.5 and cell surface Tetherin-HA measured with anti-HA followed and flow cytometry. Cells transduced (TagRFP+ population) with a viral protein expression vector (solid line) and empty lentiviral vector (dotted line) were gated and compared. Representative of two experiments.

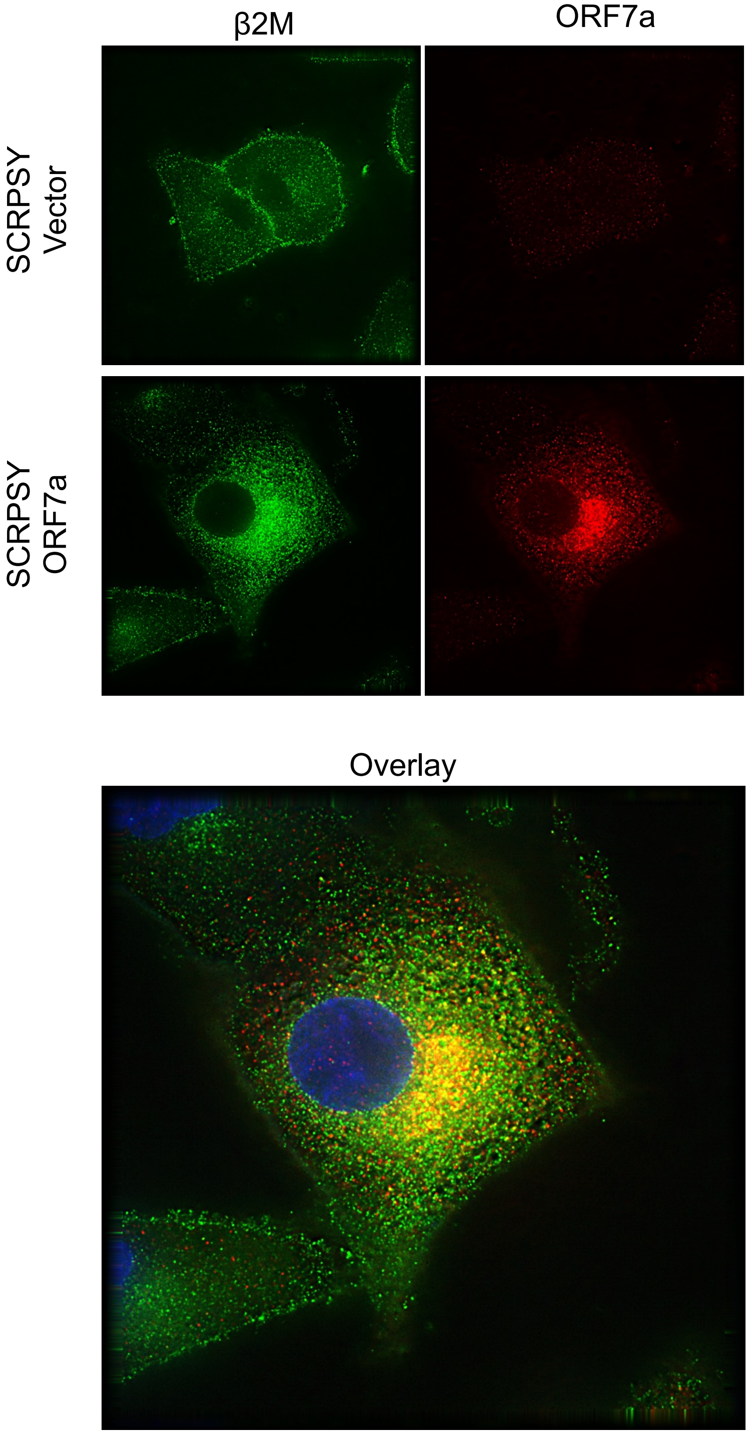

Figure S2 Redistribution of β2M in SARS-CoV-2 ORF7a-expressing cells. Immunofluorescent staining of A549 cells with anti-β2M (green) and anti-ORF7a following transduction with empty SCRPSY (vector) or ORF7a-expressing SCRPSY (ORF7a). A single optical section following deconvolution microscopy is shown.

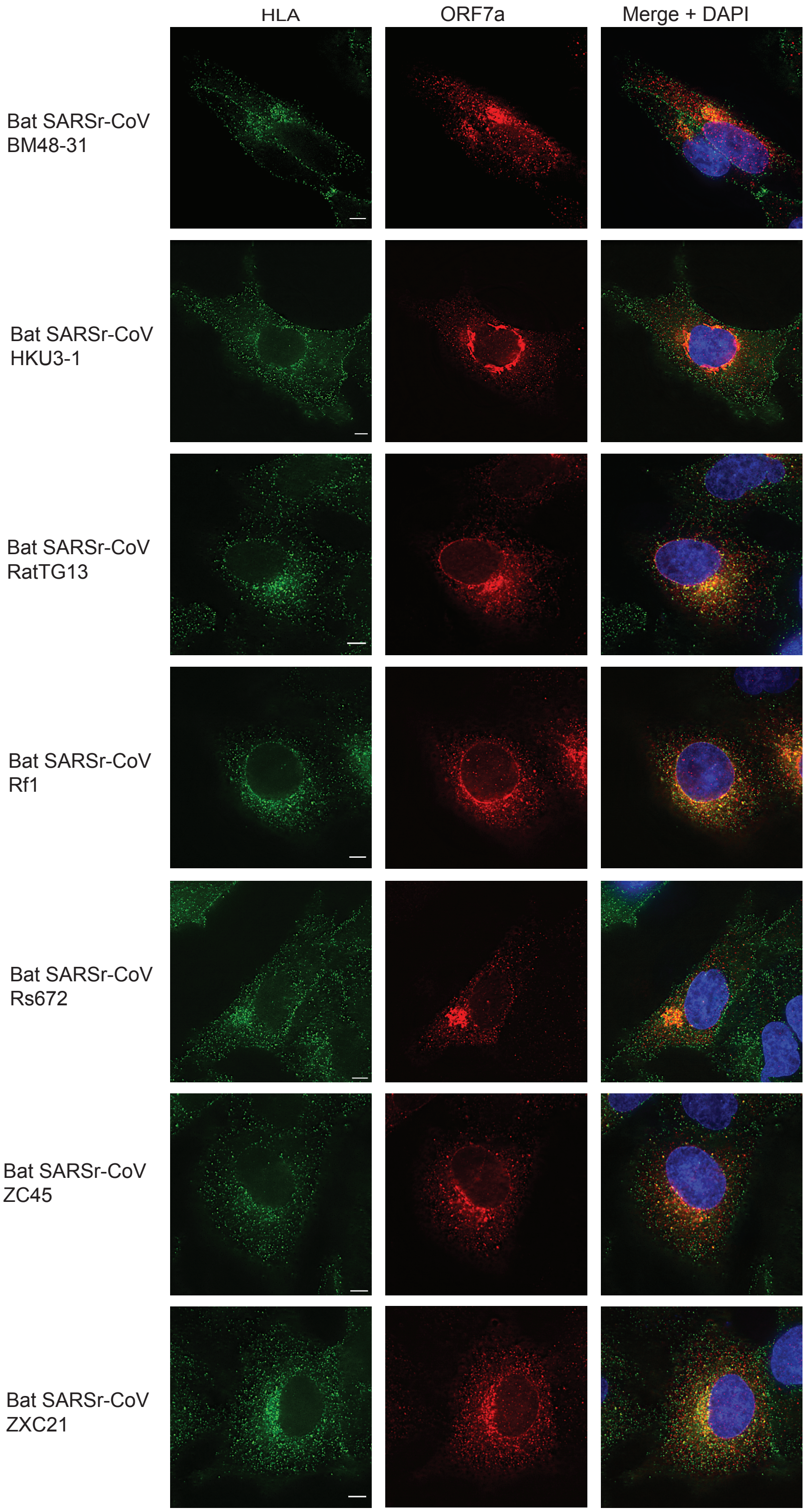

Figure S3 Distribution of MHC-I in bat SARSr-CoV ORF7a-expressing cells. Immunofluorescent staining of A549 cells with anti-HLA-A (green) and anti-ORF7a (red) following transduction with SCRPSY (vector) or SCRPSY expressing ORF7a from the indicated SARSr-CoV. A single optical section following deconvolution microscopy is shown.

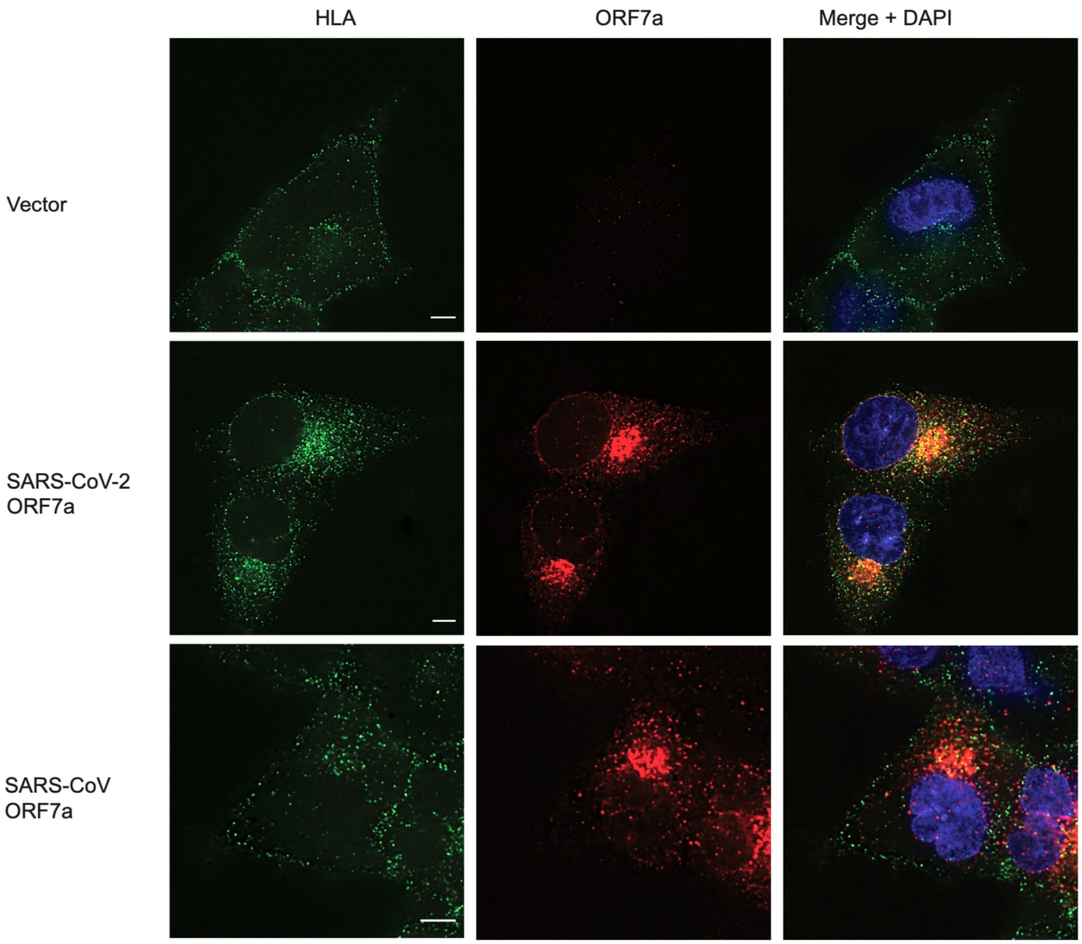

Figure S4 Distribution of MHC-I in SARS-CoV-2 or SARS-CoV ORF7a-expressing cells. Immunofluorescent staining of A549 cells with anti-HLA-A (green) and anti-ORF7a (red) following transduction with SCRPSY (vector) or ORF7a-expressing SCRPSY. A single optical section following deconvolution microscopy is shown.

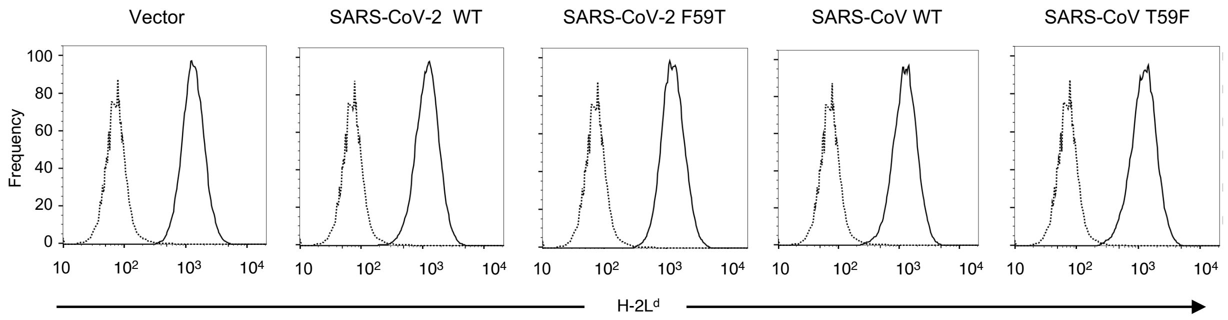

Figure S5 Lack of MHC-1 downregulation by ORF7a in NIH3T3/CycT1 cells.
NIH3T3 cells engineered to express humanized CycT1 to enable efficient expression by the SCRPSY vector were transduced with pSCRPSY-based lentiviral vector expressing SARS-CoV-2, or SARS-CoV ORF7a at an MOI of 0.5. Cells were then stained with isotype IgG (dotted line) or an anti-mouse MHC-I (H-2L^d) antibody (clone 30-5-7S) (solid line) followed by flow cytometry. Cells transduced (TagRFP+ population) with the ORF7a expression vectors or the empty lentiviral vector were gated and compared. Representative of three experiments.

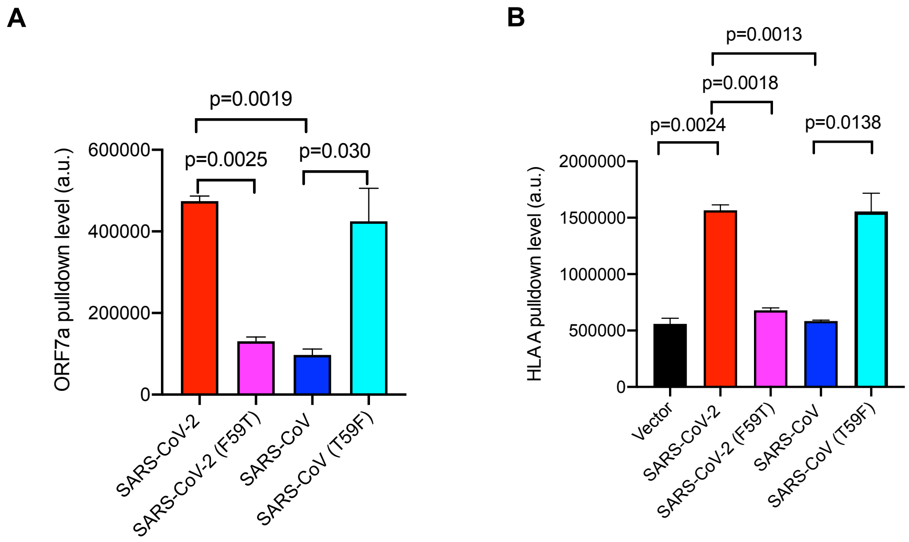

Figure S6 Western blot quantification of co-immunoprecipitated ORF7a and MHC-I
(A) Western blot quantification of ORF7a in immunoprecipitates (IP) as indicated in Figure 4A. The mean and range of values from two independent experiments are plotted. P-values are calculated using the t-test with Welch's correction.
(B) Western blot quantification of HLA-A in immunoprecipitates (IP) as indicated in Figure 4B. The mean and range of values from two independent experiments are plotted. P-values are calculated using the t-test with Welch's correction.

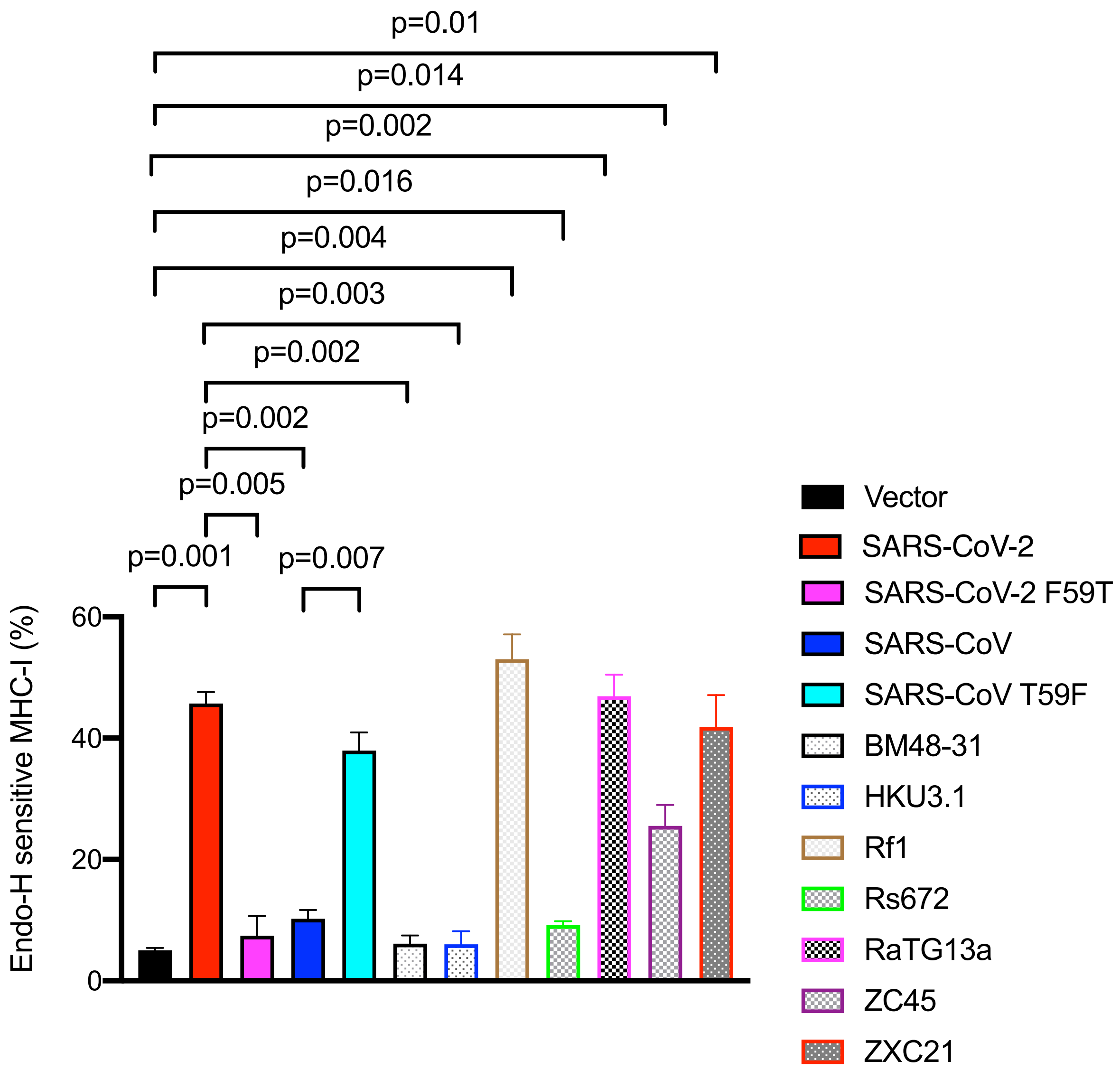

Figure S7 Quantification of Endo-H sensitive MHC-I in ORF7a-expressing cells
Cell lysates of A549 cells transduced with SCRPSY expressing no ORF7a (vector) or the ORF7a proteins from sarbecoviruses as shown in Figure 5A were subjected to Western blot quantification, from which the percentage of Endo-H sensitive MHC-I was calculated. The mean and range of values from two independent experiments are plotted. P-values are calculated using the t-test with Welch's correction.

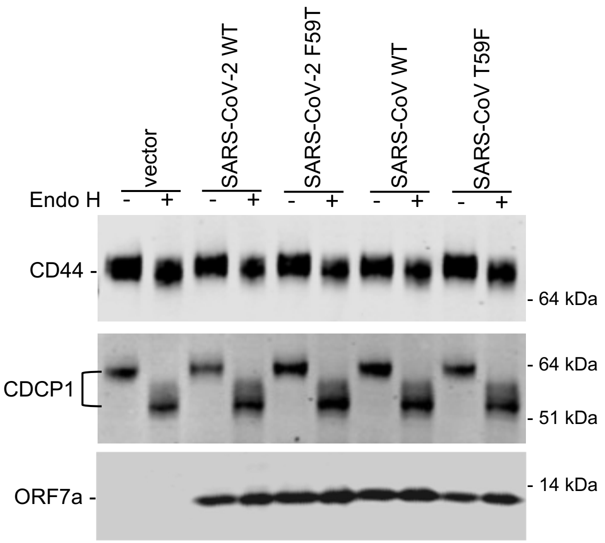

Figure S8 No evidence of effects on CD44 or CDCP1 trafficking and maturation by ORF7a.

Western blot analysis of cell lysates of A549 cells transduced with SCRPSY expressing no ORF7a (vector) or the ORF7a proteins from SARS-CoV-2, SARS-CoV (or mutants thereof) at an MOI of 0.5. Cell lysates were untreated or treated with endoglycosidase H, as indicated prior to analysis.

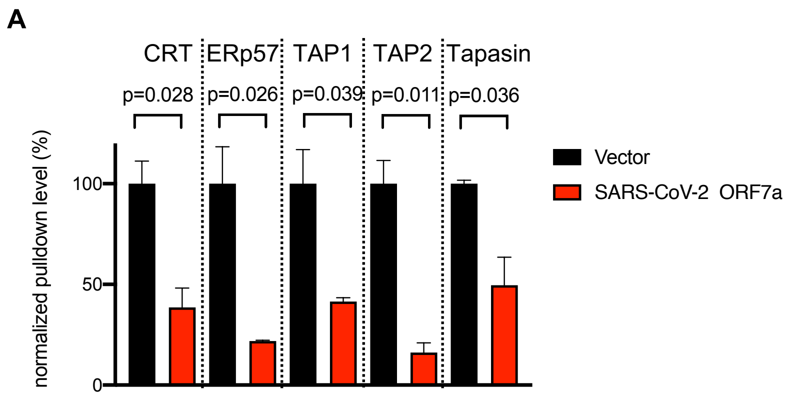

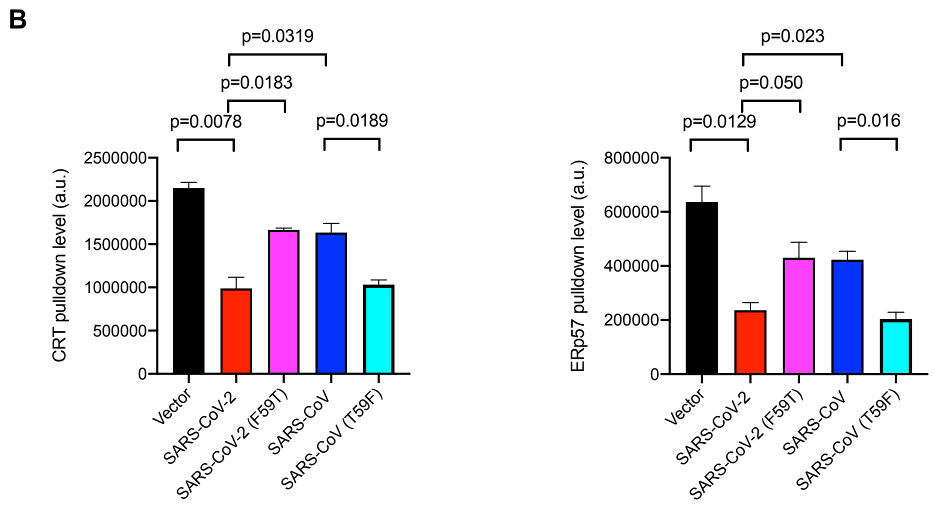

Figure S9 Western blot quantification of disruption of the MHC-I peptide loading complex assembly by ORF7a

(A) The level of CRT, ERp57, Tap1, TAP2, Tapasin in immunoprecipitates (IP) from 293T cells transduced with SCRPSY expressing no ORF7a (vector) or the SARS-CoV-2 ORF7a protein as indicated in Figure 5B was measured by Western blot quantification and normalized by the corresponding control (vector). The mean and range of values from two independent experiments are plotted. P-values are calculated using the t-test with Welch's correction.

(B) Western blot quantification of CRT (left panel) and ERp57 (right panel) in immunoprecipitates (IP) as indicated in Figure 5C. The mean and range of values from two independent experiments are plotted. P-values are calculated using the t-test with Welch's correction.

Table S1: Antibodies and reagents used in this paper

Immunoblotting (IB)

Target Antigen	Target species	Host species	Clone	Company	Catalog #
SARS-CoV / SARS-CoV-2 ORF7a		Mouse	3C9	GeneTex	GTX632602
ORF7a		Rabbit	polyclonal	Bioworld	NCP0011
HLA-A	Human	Rabbit	EP1395Y	abcam	ab52922
HLA-A, B, C	Human	Mouse	EMR8-5	abcam	ab70328
CD44	Human	Rabbit	polyclonal	abcam	ab243894
CDCP1	Human	Rabbit	polyclonal	ThermoFisher	PA5-17245
Calreticulin	Human	Rabbit	polyclonal	abcam	ab2907
ERp57	Human	Rabbit	polyclonal	abcam	ab10287
TAP1	Human	Rabbit	polyclonal	ThermoFisher Proteintech	11114-1-AP
TAP2	Human	Rabbit	polyclonal	abcam	ab180611
TAP2	Human	Rabbit	polyclonal	ThermoFisher Invitrogen	PA5-37414
Tapasin	Human	Rabbit	polyclonal	ThermoFisher Invitrogen	PA5-42731
Tapasin	Human	Mouse	TO-3	Santa Cruz	sc-80647
Tubulin	Human	Mouse	DM1A	Sigma	T9026

Flow cytometry (FCM)

Target Antigen	Target species	Host species	Clone	Conjugation	Company	Catalog #
HLA-A, B, C	Human	Mouse	W6/32	AF488	Biolegend	311413
HLA-A, B, C	Human	Mouse	W6/32	AF647	Biolegend	311414
HLA-A2	Human	Mouse	BB7.2	APC	Biolegend	343308
HLA-A	Human	Rat	YTH 862.2		abcam	Ab00200-8.1
H-2L^{d}	Mouse	Mouse	30-5-7S	AF488	Cedarlane	CL9011AF4
HA epitope	tag	Mouse	16B12	AF488	Biolegend	901509
HA epitope	tag	Mouse	16B12	APC	Biolegend	901524

Immunoprecipitation (IP)

Target Antigen	Target species	Host species	Clone	Company	Catalog #
SARS-CoV / SARS-CoV-2 ORF7a		Mouse	3C9	GeneTex	GTX632602
HLA-A, B, C	Human	Mouse	W6/32	Biolegend	311402
HLA-A, B, C	Human	Mouse	5C5B7	Proteintech	66013-1-Ig
HLA-B	Human	Rabbit	polyclonal	ThermoFisher Invitrogen	PA5-35345

Immunofluorescence (IF)

Target Antigen	Target species	Host species	Clone	Company	Catalog #
SARS-CoV / SARS-CoV-2 ORF7a		Rabbit		Bioworld	NCP0011
HLA-A, B, C	Human	Mouse	W6/32	Sigma	H1650-100TST
HLA-A	Human	Rabbit	EP1395Y	Abcam	ab52922
B2M	Human	Mouse	B2M/961	Novus	NBP2-44471

IgG H+L	Rabbit	Goat		Novus	NBP1-72732C
IgG H+L	Mouse	Goat		Novus	NBP1-72739JF646
IgG H+L	Mouse	Goat		Thermo	A11029

Dataset S1
Nucleotide sequences of codon-optimized SARS-CoV-2 ORFs
